# Sphincteroplasty of the Sphincter of Oddi in the Treatment of Benign Distal Obstructions of the Bile Duct

**DOI:** 10.1155/1989/74538

**Published:** 1989

**Authors:** S. Duca

**Affiliations:** 1 Surgical Clinic III of the Faculty of Medicine Cluj-Napoca Romania; 2 str. Firiza nr. 2 apt. 17 Cluj-Napoca 15 R – 3400 Romania

## Abstract

Seventy patients in whom sphincteroplasty was performed by an original technique are presented. In 65 cases the indication was stenosis of the sphincter of oddi, associated or not with cholelithiasis or hepatic hydatid disease. There were relative indications in another 5 patients. Sphincteroplasty was achieved with the aid of an original probe, and average length of the incision of the ampullary area was 28 mm.

In the immediate postoperative period there was one case of acute postoperative pancreatitis, one duodenal fistula and an upper digestive haemorrhage; also a residual stone was detected. All these complications have responded favourably to conservative treatment. There was a single death in an old patient with bronchopneumonia.

The late results were very good or good with the exception of two cases: one which presented with cholangitis episodes maintained by duodenal stasis, and one female patient, who after one year from sphincteroplasty had to be reoperated on for an hepatic abscess.


*HPB Surgery* 1989, Vol. 1, pp. 131–140
Reprints available directly from the publisher
Photocopying permitted by license only

© 1989 Harwood Academic Publishers GmbH
Printed in Great Britain

### SPHINCTEROPLASTY OF THE SPHINCTER OF ODDI IN THE TREATMENT OF BENIGN DISTAL OBSTRUCTIONS OF THE BILE DUCT

### A Prospective Study of 70 Cases managed by a Original Surgical Technique

S. DUCA*

*Surgical Clinic III of the Faculty of Medicine, Cluj-Napoca, Romania*

*(Received 17 August 1988)*

Seventy patients in whom sphincteroplasty was performed by an original technique are presented. In 65 cases the indication was stenosis of the sphincter of oddi, associated or not with cholelithiasis or hepatic hydatid disease. There were relative indications in another 5 patients. Sphincteroplasty was achieved with the aid of an original probe, and average length of the incision of the ampullary area was 28 mm.

In the immediate postoperative period there was one case of acute postoperative pancreatitis, one duodenal fistula and an upper digestive haemorrhage; also a residual stone was detected. All these complications have responded favourably to conservative treatment. There was a single death in an old patient with bronchopneumonia.

The late results were very good or good with the exception of two cases: one which presented with cholangitis episodes maintained by duodenal stasis, and one female patient, who after one year from sphincteroplasty had to be reoperated on for an hepatic abscess.

KEY WORDS: Oddian sphincteroplasty, original technique, early and late results.

### INTRODUCTION

In spite of their apparent similarities, sphincterotomy (ST) and sphincteroplasty (SP) should be seen as two different procedures. The 10 to 18 mm incision performed in ST spares a part of the sphincter of oddi and the newly created aperture maintains its contractile character. By contrast, in SP – as described by Jones[1], the incision is 27 to 30 mm long, completely suppressing the function of the sphincter mechanism and a non-contractile opening with a diameter equal to that of the common bile duct (CBD) is obtained. This fact favours the spontaneous elimination of residual stones and reduces the risk of restenosis.

The author's personal experience in this field, during the last seven years, using an original technique for SP[2,3], is presented below.

* Please direct all correspondence to: Dr. sc. med. Sergiu Duca, str. Firiza nr. 2, apt. 17 R – 3400 Cluj-Napoca 15, Romania.

### MATERIAL AND METHOD

### *1. Clinical material*

SP was performed in 70 patients (7.4%) from a total of 941 biliary operations which represents a personal series from 1981 to 1987. The patients ranged in age from 28 to 75 years (mean age, 51.4 years). Twenty one of the 70 patients were men and 49 were women. There were absolute indications in 65 cases of benign stenosis of oddi (Table 1). There were relative indications in 5 cases, where the aim was to provide the CBD with a wide drainage channel.

**Table 1** Intraoperative diagnosis

| | cases |
|---|---|
| 1. Absolute indications (92.8%) | 65 |
| Primary oddian stenosis | 1 |
| Stenosis of the sphincter of Oddi (SSO) + gallbladder stones | 26 |
| SSO + gallbladder stones + stones in the CBD | 13 |
| Stone impacted in papilla | 4 |
| SSO + cholecysto-choledochal fistula | 2 |
| SSO + hydatid cyst ruptured into the bile ducts | 4 |
| Residual SSO | 10 |
| Residual SSO + stones in the CBD | 5 |
| 2. Relative indications (7.2%) | 5 |
| Choledochal microlithiasis | 3 |
| Hydatid cyst ruptured into the bile ducts | 1 |
| Stump syndrome after choledochoduodenostomy | 1 |
| TOTAL | 70 |
| Primary interventions (77.1%) | 54 |
| Biliary reinterventions (22.9%) | 16 |

The diagnosis of the sphincter stenosis was mainly based on operative assessment. We performed peroperative cholangiography in all cases. The simultaneous administration of cholecystokinin permitted the discrimination of spasm from stenosis. However the reversibility of the lesion was determined by instrumental exploration of the papilla: when a rubber probe of diameter 3 mm would not pass the papilla, we considered this an indication for SP[1–6]. In the case of a large or dilatable cystic duct, we performed this exploration via the cystic blunt (21 cases). However in the majority of cases, a choledochal opening was necessary (49 cases).

### *2. Personal Procedure for Sphincteroplasty*

A. The probe for sphincteroplasty (Figure 1): This comprises a metallic portion formed by three thin branches 30 mm in length, which form an approximately ellipsoidal shape (b). On transverse section (e) the branches are located corresponding to the hours 10, 2 and 6. A short button-tipped rod (a), 10 mm long, arises from the distal end. Another thicker hollow tube (c) 3 mm long, arises from the proximal junction of the branches, this is connected to a plastic tube (d), 20 to 25

cm long. The flexibility of this tube allows easy insertion of the probe into the CBD without risking the creation of a false passage. In addition, by applying suction to the tube, removal of blood and secretions is possible, so that no other instrument need be introduced into the surgical field.

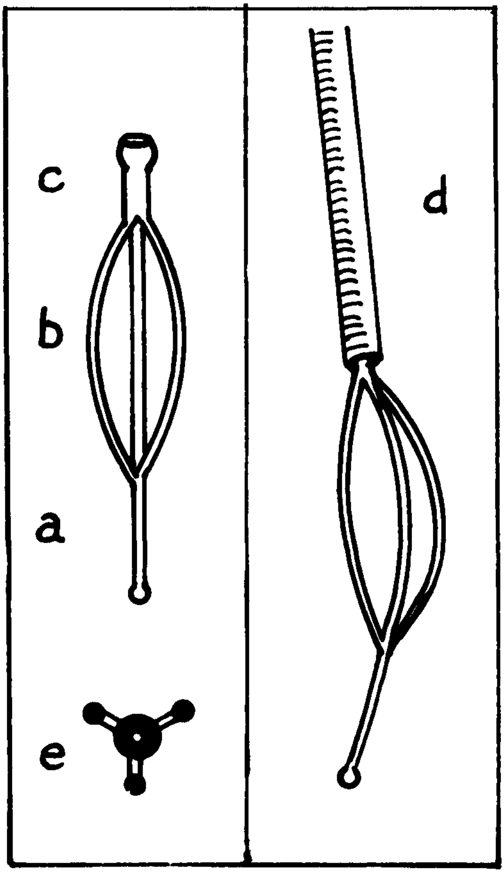

**Figure 1** The probe for sphincteroplasty.
a. Button-tipped rod; b. Ellipsoidal segment; c. Rod provided with an axial channel; d. Plastic tube; e. Transverse section.

The metallic portion describes a moderate curve with an anterior concavity which allows easy passage into the inferior segment of the CBD. In order to provide optimal conditions in the use of the probe, two variants with transverse diameters of the ellipsoidal section of 7 and 9 mm were constructed.

B. Surgical procedure: The papilla is detected by introducing the probe into the CBD through a supraduodenal choledocochotomy (Figure 2). The button-tipped rod passes the papilla even in cases of tight stenosis and it can be felt through the anterior duodenal wall thus indicating the exact site for transverse duodenotomy (10–15 mm). By pulling the button-tipped rod, the papilla is brought into the operative field (Figure 2 – insert).

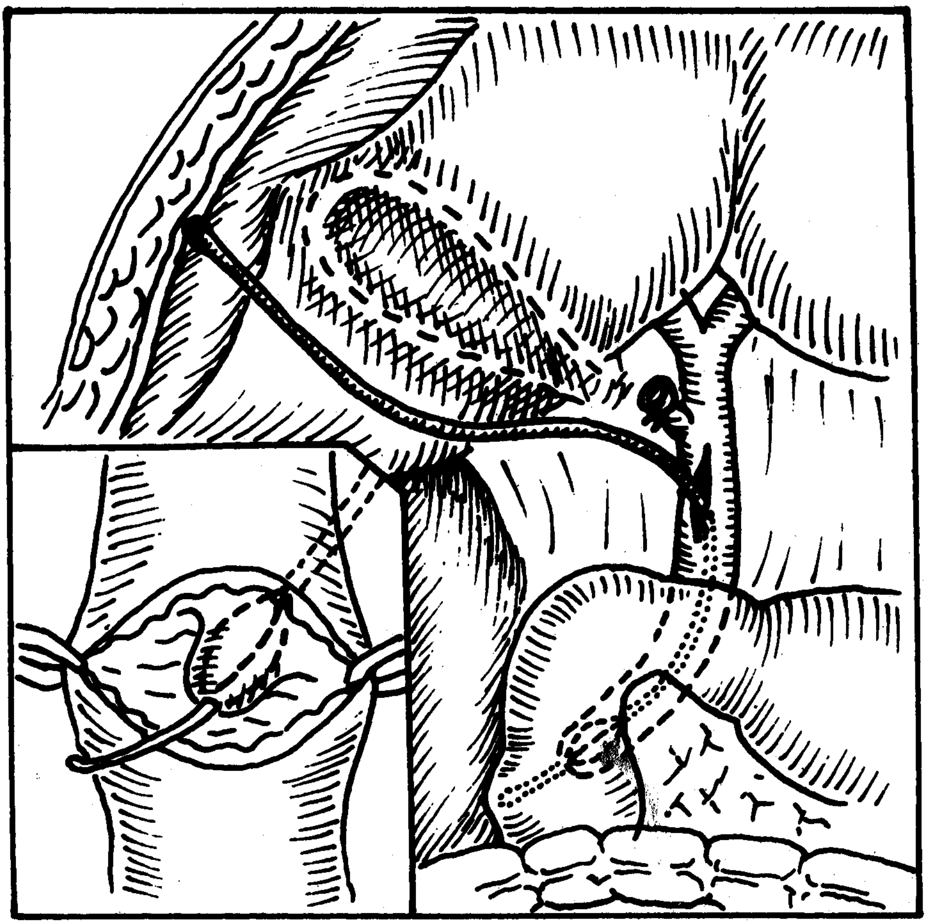

**Figure 2** Papilla is detected by introducing the probe into the inferior segment of the common bile duct.

We start by incising the anterior wall of the infundibulum which is spread on the two superior branches of the metallic portion of the probe. An opening of 10 to 15 mm is made using fine scissors and taking care to avoid cutting through the papillary ring. This detail is very important, since the infundibulo-papillary region remains elevated into the operative field only for as long as the papillary ring is suspended by the button-tipped rod of the probe (Figure 3A). We consider that this is one of the major advantages of our method because the papillary ring – the element of maximal anatomical resistance – provides a support and lessens the risk of damaging this rather delicate region.

The moment the opening is made, separation of duodenal mucosa from that of the CBD occurs. Before extending the incision upward, 2 or 3 pairs of 3–0 catgut sutures are inserted on each sides of the incision. Particular attention should be paid to the closure of the incision's superior angle: if an accidental communication with

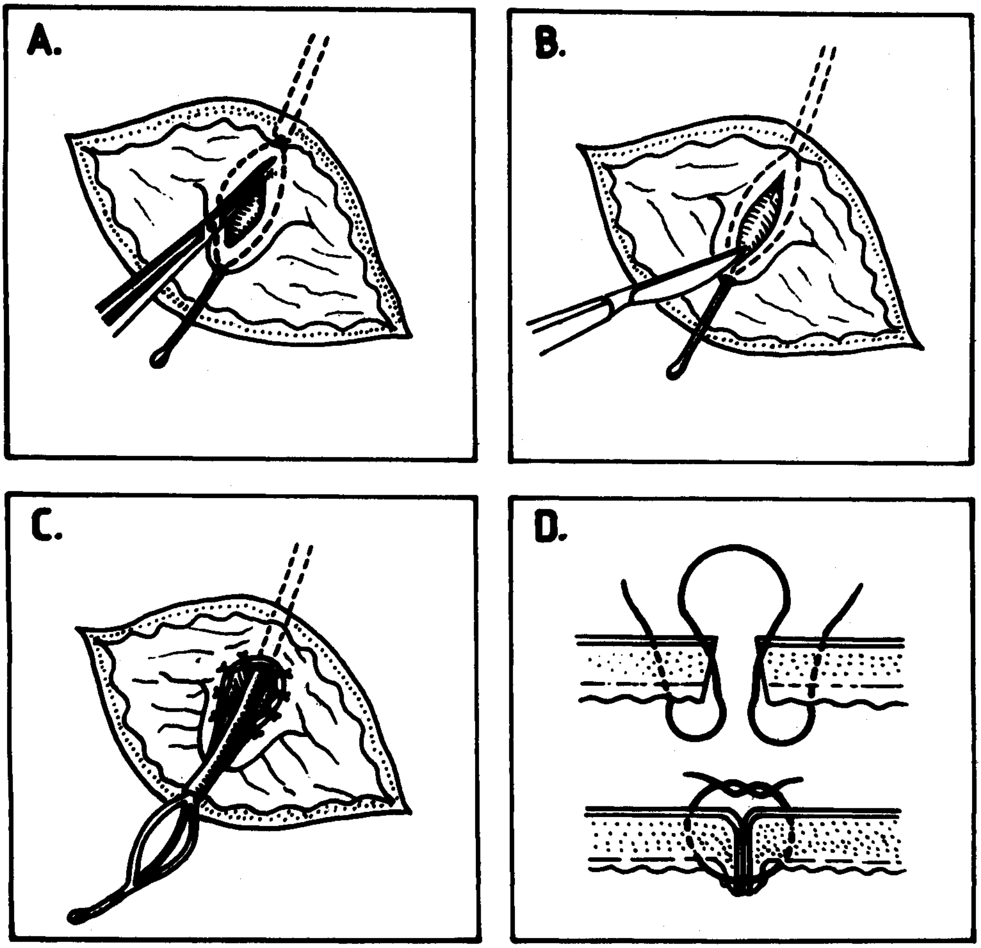

**Figure 3** A. Incision of the anterior wall of the infundibulum. B. Incision of the papillary ring. C. The sphincteroplasty is performed; the probe passes easily into the duodenum. D. Closure of the duodenum by means of the omega shape thread.

the retroperitoneal space occurs, Jones[1] recommends the use of an "8" shape stitch.

Once the infundibulum is opened, the plastic tube is connected to a suction outlet ensuring the removal of blood from the operation field. Bleeding can be substantial in this region.

The length of the probe's ellipsoidal portion (30 mm) provides a guide for measuring the length of the incision. As a rule, with an incision of 25 to 28 mm, a permanently open stoma, with a diameter equal to or larger than that of the supreduodenal CBD was obtained. It should be noted, however, that in some cases the intramural portion of the CBD is less than 6 mm, so that pancreatic damage may occur even when a 10 mm sphincterotomy is made[1].

The sphincteroplasty is terminated by sectioning the papillary ring (Figure 3B). As a result the whole sphincter region slips into the depths of the operative field and the metallic portion of the probe passes into the duodenum (Figure 3C).

The effect of the surgical intervention can be assessed by moving the probe up and down the bile duct. The ellipsoidal segment should pass easily through the newly created orifice, which should remain permanently open.

Catheterisation of the duct of Wirsung is not necessary since the inferior branch of the ellipsoidal segment pushes the pancreatic duct away, thus reducing the risk of its involvement in the sutures applied on the incision's margins. However, at the end, the identification of the orifice would seem to be a sensible manoeuvre to detect any narrowing.

Closure of the duodenum was performed by us, in the great majority of cases, by means of our personal technique, the "omega shaped stitch" (Figure 3D). This suture provides good haemostasis and perfect apposition of the serous layers. The duodenal lumen was thus less diminished than by a two-layer suture and the closure was stronger than after a simple one-layer suture.

The common duct was drained by a T-tube and a drain was left in the subhepatic region.

### RESULTS

1. *The immediate postoperative results* were assessed on the basis of the clinical course, radiological investigation and laboratory data.

A. During the post operative period, five complications (7.1%) were noted, three (4.2%) of them due to handling of the papilla or the duodenotomy:

One female patient developed postoperative acute pancreatitis. In this case we had found a short intraparietal choledochus as described by Jones[1]. Our incision of 10 mm damaged the pancreas. After intensive treatment, the patient was discharged well on the 28th day after surgery.

In another patient, melaena developed on the sixth day after surgery; its cause remained obscure. Haemorrhage may originate not only from the margins of the papillary incision but also from the margins of duodenotomy or from the CBD mucosa damaged by the exploration.

Complications connected with the duodenotomy were noted only in one patient aged 73, who developed a duodenal fistula. Healing occured after 14 days of therapy consisting of continuous aspiration through the subhepatic drain, total parenteral nutrition and anticholinergic drugs.

Among other complications we mention one case of residual lithiasis (finally the stone was spontaneously eliminated through the SP), and bronchopneumonia that caused the death of a 75 year old patient.

B. Radiological investigation: Cholangiography through the T-tube was performed on the tenth day following surgery. A large communication between the terminal portion of the CBD and duodenum was usually seen. Like Pichlmayr[7] we found that – in spite of a precise and extended incision – the flow of contrast medium is sometimes surprisingly poor. In a single case we noted the presence of a residual stone in the lower CBD.

C. Laboratory investigation: We perform regular determinations of urinary amylase by the photometric (amyloclastic) method (modified Richterich procedure; normal levels in urine up to 8,000 I.U.). Increased urine amylase activity was found in 31 patients (44.2%). Excepting one case with the clinical signs of acute postoperative pancreatitis, any inflammation of the pancreas remained at the preclinic stage with the urinary amylase moderate levels (ranging from 15,000 I.U.

to 60,000 I.U., the average of the maximal levels being 18,433 I.U.). The period of urine amylase activity lasted from 6 to 21 days (average 7 days).

2. *Late postoperative results:* The patients were submitted to periodic checks for between 3 months and 7 years. In order to assess the late results, we have applied Visick's scale[8]. 67 patients in whom good resulted were noted, were included in grades 1 and 2. Poor results were found in two patients. One of them (Visick grade 3), suffered two episodes of cholangitis needing treatment with antibiotics; the barium contrast X-ray revealed that besides the duodeno-choledochal reflux (usual after SP), there was duodenal stasis. This was satisfactorily treated with metaclopramide. In grade IV (unsatisfactory results), we include the same female patient who had postoperative acute pancreatitis, one year after sphincteroplasty surgical drainage of an hepatic abscess was required.

Serum bilirubin and alkaline phosphatase levels were followed in all patients and remained normal. Other investigations (ultrasonograph, biliary scintigraphy, barium contrast X-ray), were performed exceptionally in the patients with a Visick's grade 3 or 4..

### DISCUSSIONS

Operations on the papilla present one of the most hotly disputed controversies in the history of surgery.

1. *Indications for operation* some surgeons have denied the existence of stenosis of the sphincter of oddi. This view gave papillary surgery the reputation of being meddlesome. However, clinical practice proved that not accepting the existence of stenosis or resistance to operate adequately on the papilla – entails a risk of revisional surgery in this area. Thus Safrany[9] in 342 patients with painful post-cholecystectomy syndrome, explored by retrograde endoscopic cholangiography has found in 164 cases residual sphincter stenosis. During biliary revisional surgery, also these lesions were found in 17% to 60% of cases[6,7,10,11]. Therefore it is implied that during the primary operation sphincter surgery is performed in fewer cases than is clinically necessary[10].

The progress recorded in the last decade for endoscopic sphincterotomy (ES), has promoted new disputes concerning the indications for procedures on the papilla. The enthusiasm for the endoscopic method has paradoxically seduced those who have considered stenosis to be a "myth" and sphincterotomy an excess. With only a few exceptions, the complete correction of the biliary lesions during the primary intervention remains as the first duty of the surgeon. ES is to be performed only in patients at very high risk and especially in those over 65 years of age. Were, the death rate due to the surgical method (over 5%) will be reduced to below 2%[12].

In so far as failure of biliary surgery is concerned, we have to admit that ES represents the most elegant and the most efficient means of correction. In our data second operations on the papilla account for 22.9% of all cases. We are convinced, however, that the conventional ST will have a more restricted application when the endoscopic method has become more widely available.

2. *The risks and the therapeutic benefits of papillary surgery.* This represents the second aspect that we intend to discuss.

In spite of its apparently simplicity incision of the sphincter of oddi is an intervention requiring the utmost precision and finesse, which naturally implies an element of risks also. In the early days of papillary surgery, the occurrence and severity of postoperative complications was source of such fear that the controversies over the usefulness and value of these surgical procedures cannot yet be judged as settled. It is obvious that no one can deny the risks involved in either ST or SP, but these risks should not cause us to give up but to be cautious. On the other hand, an appreciation of the operative indications and the refinement of the surgical technique attained during the last decade, have significantly decreased the rate of failure. Thus, an international inquiry initiated by Negro[13] on 25,541 cases operated on by 150 surgical teams in 50 countries, had revealed 2.3% morbidity and 1.8% global mortality (0.8% due to specific causes). These data prove that highly trained hands are able to manage procedures on the sphincter of oddi without more severe or more frequent complications than occur after other operations performed on other sections of the digestive tract in general and on the biliary tree in particular.

The most fearsome of complications is, undoubtedly, postoperative acute pancreatitis (PAP). In some work it is estimated at 3 to 5%[14–18]. Generally, all workers agree that in biliary surgery manipulation of the papilla has the highest potential for triggering PAP. But one important contribution is manoeuvres having a traumatic effect on the papilla, associated either with the exploring stage or with the surgical act as such. Their importance has and still is ignored and, as Leger[19] put it, in spite of these traumatic manoeuvres, the complications are ascribed to the final act of sphincterotomy and not to the preceding aggresion, attributing to the sphincter what belongs in fact to the surgeon (B. Roy cited by[19]). By practising as non-traumatic surgery as possible allows for a reduction in the occurrence of this complication (0.59% of cases in Negro's[13] international inquiry). Our own statistics shows its occurrence in only one female patient (1.4%) who displayed the short intramural choledochus in which, along with the incision of the papilla, the pancreatic tissue was damaged. In an other 30 patients moderate urine amylase hyperactivity was found, but any pancreatitis was at a preclinic stage (Hilario's[18] "laboratory pancreatitis"). Without minimalizing the risk of PAP, we consider that the paralysing fear of this possible complication cannot justify abstention from surgery on the papillary region when the indication is correct.

Duodenal fistula are in second place for causes of death, after surgery of the papilla. It is however a rarely occurring complication (0.55% according to Negro[13] and 1.4% in our statistics) and it is slightly more frequent after large longitudinal duodenomoties than after the transverse ones[13]. When it has a supravaterian position, the conservative treatment had good chance of success, a fact proved by our observations.

One still widely disputed problem is that of the duodene-choledochal reflux produced by SP. It can be detected in barium contrast X-rays by the passage of contrast medium into the CBD (Figure 4). This fact, according to Jones[1], demonstrates the permanently open nature of the stoma and so, the efficiency of SP. The opponents of the method maintain that the alimentary reflux from the duodenum is one of the great drawbacks of SP. But the reflux does not cause, by itself, the development of postoperative cholangitis; this pathological condition is dependent on factors which hamper efflux and therefore generate stasis. In other words, the infection is not ascending but descending. In our clinical study only one patient showed the symptoms of cholangitis; this was caused and maintained by duodenal stasis and required, besides antiobiotics, the treatment of dyskinesia. We did not find any other or more frequent causes for stasis and biliary infection, as for example, restenosis of the papilla or residual stones. This is due to the SP ensuring

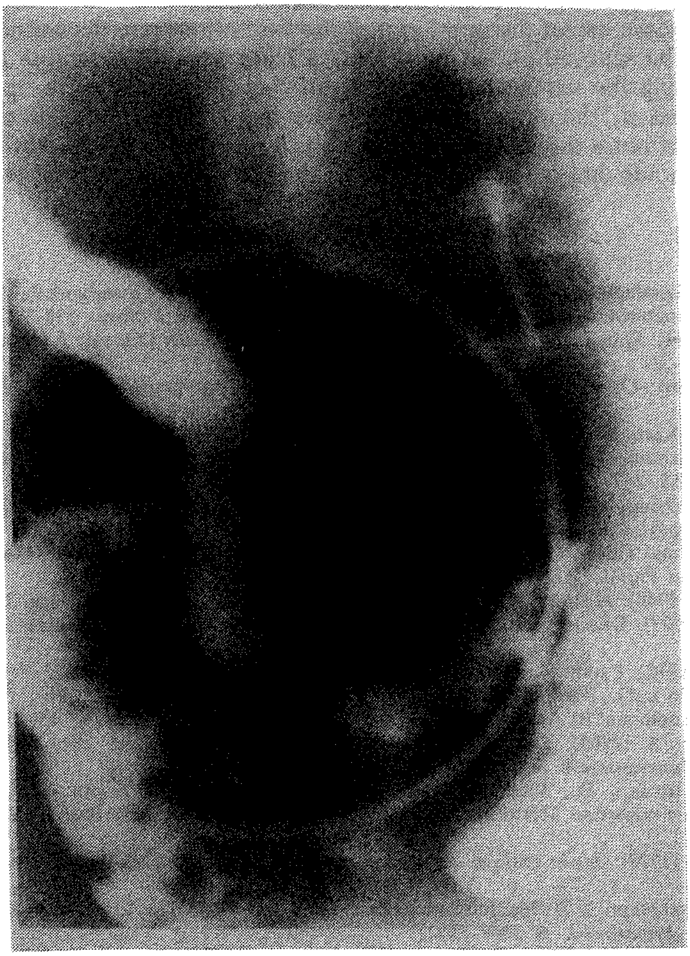

**Figure 4** Massive reflux of barium contrast medium into the bile ducts (three months after sphincteroplasty).

good drainage of the CBD and avoiding these complications which are more frequent after ST.

These results show that when an adequate technique is used and when the indications for the method are adhered to, complications in the surgery of the sphincter of Oddi may largely be avoided. Moreover, most authors report good or very good results, i.e. 75 to 90% of the cases[1,3,5–7,10,11,13,14,16–18,20,21], and the late postoperative checks revealed that neither the bile ducts nor the liver were damaged by the intervention[1,7,10,11,20]. Our case history reveals that the only serious problem was that of the female patient who had to undergo drainage of a liver abscess after SP (successive checks have ceased to reveal any trouble).

Death rate is also low after the operation on the papilla – 1.8% (0.8% due to specific causes) in Negro's[13] statistics. Our own data reveal a single death by a pulmonary complication (1.4%).

In conclusion, we are of the opinion that the vast experience accumulated so far can no longer be ignored and it represents a clear argument against those who persist in denying the value and usefulness of the operations on the papilla. As Stoppa[21] has put it, the time of controversies has run out; even if the oddian sphincteromie is not a universal remedy, it is not at all the "Cinderella" of biliary surgery but it holds one of the first places in the list of CBD surgical techniques.

### *References*

1. Jones, S.A., Steedman, R.A., Keller, T.B. and Smith, L.L. (1969): Transduodenal Sphincteroplasty (not Sphincterotomy) for Biliary and Pancreatic Disease. *Am. J. Surg.*, **118**, 292–298.
2. Duca, S., (1984): Sphincteroplasty in the Treatment of Biliary Tract Disease. *Langenbecks Arch. Chir.* **360**, 25–31.
3. Duca, S. (1986): Celedocul – patologie, explorare, terapeutica chirurgicala, Ed. Dacia (Cluj-Napoca).
4. Kraus, H. and Kern, E. (1963): Bougierung des Papilla Vateri. *Dtsch. Med. Wschr.* **88**, 754–756.
5. Eichfuss, P., Schumpelick, V. and Schreiber, H.W. (1980): Benigne Papillenstenose. *Langenbecks Arch. Chir.* **351**, 267–276.
6. Köle, W. and Ornig, H. (1980): Diagnostik der Papillenstenose, Indikation und Technik der Papillotomie. *Zentralbl. Chir.* **105**, 1546–1552.
7. Pichlmayr, R. and Tidow, G. (1980): Indikatorische und technische Fehler in der Gallenchirurgie: Eingriffe an der Papille. *Aktuelle Chir.* **15**, 297–306.
8. Visick, A.H. (1948): A study of the failures after gastrectomy. Ann. R. Cell. Surg. **23**, 266–284.
9. Safrany, L. (1977): Duodenoscopyc Sphinceretomy and Gallstones Removal. *Gastroenterol.* **72**, 338–343.
10. Alnor, P.C. (1978): Papillotomie en principe. *Langenbecks Arch. Chir.* **347**, 537–544.
11. Gregg, J.A. Clark, G., Barr, C., McCartney, A., Milano, A. and Volcjak, C. (1980): Postcholecystectomy Syndrome and its Association with Ampullary Stenosis. *Am. J. Surg.* **139**, 374–378.
12. Van Der Spuy, S. (1986): The management of common bile duct stones – surgical removal or endoscopic sphincterotomy. *South African J. Surg.* **24**, 103–104.
13. Negro, P., Tuscano, D., Flati, G. and Carboni, M. (1984): Le risque opèratoire de la sphinctórotemie oddienne. Résultats d'une enquete internationale. (25.541 cas). *J. Chir.* (Paris) **121**: 133–139.
14. Salembier, Y. (1970): Etude critique de 207 interventions de sténoses oddiennes. *Ann. Chir.* **24**, 1105–1112.
15. Bismuth, H. and Hepp, J. (1976): Indications de la sphinctérotomie dans le traitement de la lithiase biliaire. Neuv. *Presse mèd.* **5**, 2949–2951.
16. Mallet-Guy, P. and Rousset, L.P. (1965): Statistique personelle de sphinctérotomie. *Rev. Int. Hépat.* **91**, 987–994.
17. Arianoff, A.A. (1980): Analysis of 607 Cases of Choledochal Sphincteroctomy. *World J. Surg.* **4**, 483–486.
18. Hilario, J. (1977): Centibuicae à cirurgia de celédoco terminal. Rio de Janeiro.
19. Leger, L. (1982): Commentaires du rapporteur. *Chir.* **108**, 155.
20. Tondelli, P., Gyr, K., Luscher, M., Schuppiser, J.P., Stalder, G.A. and Allgöwer, M. (1978): Papillotomie oder Papillenplastik? Klinische und endoskopische Spätuntersuchungen nach chirurgischer Papillenspaltung. *Helv. Chir. Acta* **45**, 687–692.
21. Stoppa. R., Verhaeghe, P., Chantriaux, J.F., Endzoua, T. and Touagh, S. (1983): Comment, avec quels résultats, quant réaliser une sphinctéretomie oddienne dans la chirurgie des lithiases de la voie biliaire principale. *Chir.* **109**, 23–26.

*Accepted by S. Bengmark on 17 August 1988.*

